# Which criteria should be used to define type 2 diabetes remission after bariatric surgery?

**DOI:** 10.1186/1471-2482-13-8

**Published:** 2013-03-28

**Authors:** Ana M Ramos-Levi, Lucio Cabrerizo, Pilar Matía, Andrés Sánchez-Pernaute, Antonio J Torres, Miguel A Rubio

**Affiliations:** 1 Department of Endocrinology and Nutrition, Hospital Clínico San Carlos, Instituto de Investigación Sanitaria San Carlos (IdISSC), Facultad de Medicina, Complutense University, Madrid, Spain; 2 Department of Surgery, Hospital Clínico San Carlos, Instituto de Investigación Sanitaria San Carlos (IdISSC), Facultad de Medicina, Complutense University, Madrid, Spain

**Keywords:** Diabetes, Diabetes remission, Bariatric surgery, Remission criteria, Morbid obesity

## Abstract

**Background:**

Comparison of diabetes remission rates after bariatric surgery using two different models of criteria.

**Methods:**

Retrospective analysis of data from 110 patients with type 2 diabetes and morbid obesity who underwent bariatric surgery, preoperatively and at 18-month follow-up. Comparison of two models of remission: 1) 2009 consensus statement criteria; 2) simple criteria using ADA’s HbA1c diabetes diagnostic cut-off values.

**Results:**

Patients’ mean ± SD preoperative characteristics were: age 53.3 ± 9.5 years, BMI 43.6 ± 5.5 kg/m^2^, HbA1c 7.9 ± 1.8%, duration of diabetes 7.6 ± 7.5 years. 44.5% of patients with previous insulin therapy. With 2009 consensus statement criteria: complete, partial and no remission in 50%, 12.7% and 37.3%, respectively; with HbA1c criteria: 50%, 15% and 34.5% in the analogous categories (p = 0.673).

**Conclusions:**

We suggest a simpler approach to evaluate diabetes remission after bariatric surgery, following the rationale of the definition of diabetes itself.

## Background

Buse et al. [1] proposed in 2009 a consensus definition of diabetes remission. These clear, although strict, criteria prompt the need to reconsider diabetes remission rates after bariatric surgery.

The objective of this study is to compare diabetes remission rates using Buse’s consensus group criteria with those obtained with a simpler definition based on the American Diabetes Association’s (ADA) glycosylated hemoglobin (HbA1c) cut-off levels used to diagnose diabetes [2], after bariatric surgery.

## Methods

Data were retrospectively analyzed from a cohort of 539 patients who underwent bariatric surgery with a preoperative diagnosis of diabetes and morbid obesity (body mass index [BMI] > 35 kg/m^2^) in a single center. Information was obtained from medical records, preoperatively, and at 18 months after surgery. Duration of diabetes, previous hypoglycemic treatment, age, weight, height, BMI, fasting glucose (FG) and HbA1c were recorded. All patients signed a written informed consent prior to surgery in which it was specified that clinical and analytical data collected before the bariatric procedure and during follow up could be potentially used in an anonymous way for investigation and publication. This study was approved by the Ethics Committee of the Hospital Clinico San Carlos and was in compliance with the Helsinki Declaration.

Two criteria were used to define diabetes remission: 1) 2009 consensus statement (model 1): complete remission if “normal” measures of glucose metabolism were achieved (HbA1c < 6% and FG < 100 mg/dl [< 5.6 mmol/l]; partial remission if HbA1c < 6.5% and FG 100–125 mg/dl (5.6-6.9 mmol/l), in both cases in the absence of pharmacologic therapy or ongoing procedures, for a duration of at least one year [1]. 2) “HbA1c criteria”, based on HbA1c levels used to define diabetes in current ADA guidelines [2] (model 2): remission if HbA1c < 5.7%, improvement if HbA1c 5.7 - 6.5%, in both cases without hypoglycemic treatment and a duration of at least one year, and no remission if these criteria were not met.

Measurement of HbA1c was carried out in blood samples collected with EDTA 3 K, by using high performance liquid chromatography (HPLC) (Tosoh G8^®^). This method is monthly evaluated by the Program of External Guarantee of Quality of the Spanish Society of Clinical Chemistry, and is in compliance with ADA recommendations [2], which specify that the diagnostic test should be performed using a method that is certified by the National Glycohemoglobin Standardization Program (NGSP).

Three types of bariatric surgery were performed: laparoscopic Roux-en-Y gastric bypass, biliopancreatic diversion and sleeve gastrectomy. Eligibility for each of them varied according to the patients’ previous diabetes medical history and comorbidities.

Preoperative features were compared with the 18-month follow-up ones using *t* test for paired samples and Wilcoxon’s test. The number of patients in each remission category 18 months after surgery using both criteria was compared using Chi-square test. Preoperative characteristics were evaluated in their association with rates of the three remission categories with Kruskal Wallis and Chi square tests.

## Results

We obtained data from 110 patients with type 2 diabetes (61.8% women). Mean ± SD preoperative characteristics were: age 53 ± 10 years, BMI 43.6 ± 5.5 kg/m^2^, FG 165.2 ± 58.5 mg/dL and HbA1c 7.9 ±1.8%. Mean duration of diabetes was 7.6 ±7.5 years and 44.5% of patients were on insulin therapy prior to surgery.

At 18-months follow-up, mean ± SD BMI, FG and HbA1c were 29.0 ± 5.0 kg/m^2^, 100.2 ± 23.9 mg/dL and 5.4 ± 0.7%, respectively, all being significantly different from their corresponding preoperative values (p < 0.001 in all cases).

Eighteen months after bariatric surgery, according to model 1, 50% obtained complete remission, 12.7% partial remission, and 37.3% no remission. With model 2, rates in the analogous categories were 50%, 15% and 34.5%, respectively (Table 1). No significant differences were found whichever the criteria used (p = 0.673). Age and preoperative BMI did not influence the rate of remission. Percentage of body weight loss (% WL) and percentage of excess body weight loss (% EWL) were greater in the complete remission group (p < 0.05). Mean duration of diabetes was significantly different across the three remission categories (p = 0.001), being greater in the non-remission group (p < 0.05). Previous insulin treatment was more frequent in the non-remission group (p = 0.000).

**Table 1:** Patients’ characteristics according to each remission category

**Variable**			**2009 Consensus group criteria (Model 1)**				**ADA’s HbA1c criteria (Model 2)**			
		**TOTAL**	***Complete***	***Partial***	***No remission***	***p***	***<5.7%***	***5.7-6.5%***	***>6.5% and/or treatment***	***p***
No of patients		110	55 (50.0)	14 (12.7)	41 (37.3)		55 (50.0)	17 (15.5)	38 (34.5)	
Age (years)		53.3 ± 9.5	51.5 ± 9.8	55.6 ± 8.0	54.9 ± 9.4	0.230	52.1 ± 9.7	52.8 ± 8.4	55.2 ± 9.7	0.299
Preop - BMI (kg/m^2^)		43.6 ± 5.5	44.3 ± 5.2	43.7 ± 4.8	42.8 ± 6.0	0.514	44.1 ± 4.9	43.6 ± 6.2	43.0 ± 6.0	0.595
18 m - BMI (kg/m^2^)		29.0 ± 5.0	28.1 ± 4.5	31.8 ± 4.9	29.2 ± 5.4	0.049	28.3 ± 4.3	30.9 ± 6.0	29.3 ± 5.4	0.193
Preop – FG (mg/dl)		165.2 ± 58.5	156.0 ± 55.3	137.8 ± 26.6	186.9 ± 64.3	0.004	153.9 ± 55.1	142.0 ± 29.5	191.8 ± 64.4	0.001
18 m – FG (mg/dl)		100.2 ± 23.9	86.7 ± 7.5	104.6 ± 6.9	118.6 ± 30.4	0.000	87.7 ± 8.9	101.5 ± 12.4	119.8 ± 30.8	0.000
Preop - HbA1c (%)		7.9 ± 1.8	7.5 ± 1.5	7.0 ± 0.8	8.6 ± 1.9	0.002	7.3 ± 1.3	7.7 ± 1.8	8.7 ± 1.9	0.000
18 m - HbA1c (%)		5.4 ± 0.7	5.0 ± 0.6	5.5 ± 0.5	6.1 ± 0.6	0.000	4.9 ± 0.5	5.8 ± 0.1	6.1 ± 0.7	0.000
%WL		33.1 ± 9.4	36.1 ± 7.9	27.1 ± 7.2	31.0 ± 10.4	0.001	35.6 ± 8.1	29.4 ± 8.3	31.0 ± 10.5	0.014
%EWL		70.7 ± 21.0	75.9 ± 17.7	57.0 ± 15.3	68.6 ± 24.4	0.007	74.9 ± 17.7	63.3 ± 22.5	68.0 ± 23.7	0.070
Duration of diabetes (years)		7.6 ± 7.5	5.1 ± 4.0	4.4 ± 4.8	11.9 ± 9.9	0.001	5.3 ± 4.3	4.6 ± 3.5	12.6 ± 10.0	0.000
Previous treatment with insulin	No	61 (55.5)	39 (70.9)	10 (71.4)	12 (29..3)	0.000	37 (67.3)	14 (82.4)	10 (26.3)	0.000
	Yes	49 (44.5)	16 (29.1)	4 (28.6)	29 (70.7)		18 (32.7)	3 (17.6)	28/73.7)	

Values show mean ± SD or number of patients and percentages (%).

“Preop” = preoperative; “18 m” = 18-month; “BMI” = body mass index; “FG” = fasting glucose; “HbA1c” = glycosylated hemoglobin; “%WL” = percentage body weight loss at 18 months; “%EWL” = percentage excess body weight loss at 18 months.

## Discussion

This study revealed that using simpler criteria based on ADA’s diabetes diagnostic HbA1c cut-off values [2] (model 2) results in remission rates comparable to those obtained with the criteria proposed by the 2009 consensus group [1] (model 1). We consider that model 2 criteria were more straightforward and of easy application, as they are based on only one biochemical parameter (the HbA1c level), in the absence of hypoglycemic treatment.

The use of Buse et al’s criteria has proved to achieve lower remission rates after bariatric procedures [3], highlighting the importance of the definition to be able to compare the outcomes of studies. Buchwald et al. [4], in 3,188 patients with type 2 diabetes, reported a 56.7-95.1% resolution rate, depending on the bariatric procedure used, and defining remission as being off diabetes medications with normal FG (< 100 mg/dl [< 5.6 mmol/l]) or HbA1c < 6%. The elevated remission rates remarked in this meta-analysis and in other following studies [5,6] have generated controversial and confounding expectations regarding true remission rates for patients with type 2 diabetes after bariatric surgery. In our series, at 18-month follow-up, complete remission was achieved in 50% of patients, in agreement with rates previously communicated [3,7,8]. Therefore, the need for uniform inclusion criteria to define type 2 diabetes complete or partial remission after bariatric surgery becomes evident and, for this reason, this issue should be addressed by scientific societies and a consensus should be reached in specific position statements.

It has recently been outlined [9,10] that diabetes duration, use of insulin, the bariatric procedure performed and, presumably, preoperative BMI, all contribute to the wide variability of remission rates reported. In the present study, regardless of the model used, longer duration of diabetes and previous treatment with insulin, were found to be associated with a lower rate of remission, a finding which is consistent with most published analyses [9,11-13]. Knowledge of these previous factors may be useful to determine how much improvement can be potentially expected after a specific bariatric procedure. For instance, in our series, in accordance to what has recently been observed [8,10,14], remission rates in patients previously on insulin therapy were around 30%, whilst rates raised up to 70% in those patients receiving only oral medication. Similarly, and also agreeing with previous works [8,9], we observed an inverse relation between diabetes duration and resolution. Thus, in future studies, differences in remission rates should be specified according to previous hypoglycemic treatments and/or diabetes duration.

## Conclusions

In conclusion, we propose simpler criteria for diabetes remission after bariatric surgery based on the ADA’s definition of diabetes. These may be of a more practical and affordable clinical application, as well as realistic regarding what outcomes to expect.

## Abbreviations

ADA: American Diabetes Association; BMI: Body mass index (kg/m^2^); FG: Fasting glucose (mg/dl); HbA1c: Glycosylated hemoglobin (%); SD: Standard deviation; %WL: percentage of body weight loss; %EWL: percentage of excess body weight loss.

## Competing interests

The authors declare no conflict of interest.

## Authors’ contributions

AMR researched, analyzed and interpreted data, and wrote the manuscript. LC and PM researched data and contributed to the discussion. AS and AT researched data and performed all bariatric procedures. MAR contributed to study conception and design, data analysis and interpretation, and reviewed and edited the manuscript. All authors gave final approval of the version to be published. All authors read and approved the final manuscript.

## Pre-publication history

The pre-publication history for this paper can be accessed here:

http://www.biomedcentral.com/1471-2482/13/8/prepub
